# Visceral Leishmaniasis Clinical Management in Endemic Districts of India, Nepal, and Bangladesh

**DOI:** 10.1155/2012/126093

**Published:** 2012-05-09

**Authors:** Megha Raj Banjara, Siddhivinayak Hirve, Niyamat Ali Siddiqui, Narendra Kumar, Sangeeta Kansal, M. Mamun Huda, Pradeep Das, Suman Rijal, Chitra Kumar Gurung, Paritosh Malaviya, Byron Arana, Axel Kroeger, Dinesh Mondal

**Affiliations:** ^1^Institute of Medicine, Tribhuvan University, Kathmandu 44168, Nepal; ^2^KEM Hospital Research Center, Pune, Maharashtra 411011, India; ^3^Umeå Center for Global Health Research, Umea University, 90187 Umeå, Sweden; ^4^Rajendra Memorial Research Institute of Medical Sciences, Patna, Bihar 800007, India; ^5^Institute of Medical Sciences, Banares Hindu University, Varanasi 221105, India; ^6^International Center for Diarrheal Diseases Research, Bangladesh (ICDDR,B), Dhaka, Bangladesh; ^7^BP Koirala Institute of Health Sciences, Dharan 56700, Nepal; ^8^Special Programme for Research and Training in Tropical Diseases (TDR), World Health Organization, 1211 Geneva, Switzerland; ^9^Liverpool School of Tropical Medicine, Liverpool L35QA, UK

## Abstract

*Background*. National VL Elimination Programs in India, Nepal and Bangladesh face challenges as home-based Miltefosine treatment is introduced. *Objectives*. To study constraints of VL management in endemic districts within context of national elimination programs before and after intervention. *Methods*. Ninety-two and 41 newly diagnosed VL patients were interviewed for clinical and provider experience in 2009 before and in 2010 after intervention (district training and improved supply of diagnostics and drugs). Providers were assessed for adherence to treatment guidelines. Facilities and doctor-patient consultations were observed to assess quality of care. *Results*. Miltefosine use increased from 33% to 59% except in Nepal where amphotericin was better available. Incorrect dosage and treatment interruptions were rare. Advice on potential side effects was uncommon but improved significantly in 2010. Physicians did not rule out pregnancy prior to starting Miltefosine. Fever measurement or spleen palpation was infrequently done in Bangladesh but improved after intervention (from 23% to 47%). Physician awareness of renal or liver toxicity as Miltefosine side effects was lower in Bangladesh. Bio-chemical monitoring was uncommon. Patient satisfaction with services remained low for ease of access or time provider spent with patient. Health facilities were better stocked with rK39 kits and Miltefosine in 2010.

## 1. Introduction

Visceral Leishmaniasis (VL) or Kala-azar has a high-disease burden in endemic districts of the Indian subcontinent (annual VL incidence ranging from 9/10,000 in Nepal to 30/10,000 pop. in India and Bangladesh) [1]. These VL endemic countries have adopted a range of public health strategies towards the elimination goal of reducing VL cases to less than 1/10,000 population by 2015 [2]. Kala-azar Elimination Programs (KAEP) aim to reduce VL morbidity and mortality by early detection, diagnosis, and treatment of VL through strengthening diagnostic and treatment facilities at peripheral health care institutions and referral services. These coupled with integrated vector management strategies including indoor residual spraying, promotion of use of insecticide treated bed nets aim to reduce VL transmission and disease burden. Despite efforts, disease transmission continues. Early detection is hindered by a surveillance system which is primarily dependent on passive reporting of patients to a public health facility. Surveillance systems rarely capture VL cases treated in the private sector. Furthermore PKDL cases escape detection through routine surveillance and contribute to continued transmission of the disease in the community [3]. Many VL cases go undiagnosed in a web of unqualified medical practitioners before they are finally diagnosed and treated for VL in the qualified private or public health sector leading to considerable delays in diagnosis and treatment [1, 4]. Recent studies show that active case detection strategies increase early VL detection and reduce delays to diagnosis and treatment [5, 6]. Early diagnosis is now feasible with the increasing use of field-based rapid diagnostic tests (rK39) to detect antibodies to recombinant antigen rK39 which are highly sensitive (range 98%-99%) and specific (range 96%-97%) [7]. Drugs available for VL treatment are limited with no new drugs under development. Historically, antimonials have been the primary line of treatment but now face emerging problems of drug resistance. Amphotericin B deoxycholate used more often as a secondary line of treatment for VL cases responding poorly to antimonials requires prolonged hospitalization and repeated biochemical monitoring. Miltefosine, the only oral drug for VL, has been recently favoured as it is highly effective (94% cure rate) with minimal side effects [8–11]. Recent research has shown single-dose liposomal Amphotericin B as well as combination drug therapy to be highly effective and safe but it will take some years before it can be adopted by country control programs.

WHO TDR has supported a research program to determine disease burden, identify, and test strategies for early detection and treatment of VL since 2006. In 2009 (preintervention phase), researchers identified VL patients using active case detection strategies. In 2010, Kala-azar Elimination Program (KAEP) staff was trained in these strategies and in VL case management according to national guidelines [12–14] accompanied by an increased procurement of diagnostics and drugs through the national program. This paper looks at the pre- and postintervention prospects and constraints of VL management from a patient and provider perspective in VL endemic districts of India, Nepal, and Bangladesh within the context of KAEP activities.

## 2. Material and Methods

### 2.1. Ethics Statement

The research program was approved by the Ethics Committees of all participating research institutes and the Ethics Review Committee of the WHO. Patients and health care providers participated in the study after providing a written informed consent.

### 2.2. Study Area and VL Treatment Policies

The study was conducted in districts of Saran and Muzaffarpur in India, Sarlahi, Mahottari, and Dhanusha in Nepal and Mymensingh in Bangladesh. The VL endemicity level in these districts varied from an annual incidence of 20–25 per 10,000 in India, 5–8 per 10,000 in Nepal, and 13–31 per 10,000 in Bangladesh. Disease surveillance has been largely passive and predominantly dependent on patients reporting to public health facilities. Antimonials as first and Amphotericin B as second line was the mainstay of VL treatment until 2009. Though Miltefosine was available in the private sector in India earlier, it was introduced free of cost through the VL elimination program as first line of treatment India in 2009. In Nepal it became available in 2007 and Bangladesh in 2010. Rapid diagnostic tests (rK39) for VL have been available in district hospitals in Nepal since 2005, at Primary Health Centers in India in 2009, and in Upazila Health Complex (sub-district hospital) in Bangladesh since 2010.

### 2.3. Identifying New Cases of VL and Training for Improved Application of National Treatment Guidelines; Preintervention and Program Intervention Phase

In 2009 (preintervention phase), four active case detection strategies (camp approach, index case approach—focal house search around known VL case, incentive approach, and blanket approach—house-to-house search) were assessed for yield of new VL cases by researchers [6]. The most cost-effective of these active case detection strategies (camp approach in high VL endemic areas and index case approach in low-to-moderate VL endemic areas) was presented to the intercountry Regional Technical Advisory Group (RTAG) and then introduced as part of the VL elimination program in 2010. Standard Operating Procedures were developed and program managers, doctors, and health workers were trained and supported by the researchers in the planning of these active case detection strategies. A total of 243 health staff was trained across all sites in active case detection strategies and VL case management as per country guidelines. The health system adopted these active case detection strategies to identify new VL cases in 2010 and reinforced the procurement of VL diagnostics and drugs in peripheral health facilities while the training program—jointly developed by national program managers and the research team and supported by German cooperation (Federal Ministry for Economic Cooperation and Development (BMZ)/Gesellschaft für Internationale Zusammenarbeit (GIZ) GmbH—focussed at the correct application of national treatment guidelines (program intervention package). A total of 126 health workers and volunteers in India, 91 in Nepal, and 26 in Bangladesh were trained in active case detection strategies and home-based VL case management.

As per the research protocol, all newly identified VL patients through active case detection in 2009 who were treated with Miltefosine were interviewed by researchers at the end of their treatment for their clinical experience, their interaction and experiences with providers using a standardized pretested questionnaire. The Indian site additionally chose to interview newly identified VL patients treated with other than Miltefosine. Whereas in 2010, all newly identified VL patients were interviewed irrespective of their drug treatment by all sites. We compare treatment patterns and patient perspectives before and after the intervention.

Patient satisfaction with provider care was rated on a 5-point scale using a shortened 18-item version of the Patient Satisfaction Questionnaire (PSQ-18) [15]. Patient satisfaction mean score (range 1 to 5) in several domains was derived with high scores reflecting high satisfaction levels. Additionally, interactions between patients and providers were directly observed in a structured manner by researchers wherever feasible.

Providers were also interviewed with a standardized questionnaire to assess their knowledge and experiences of treating VL, adherence to treatment guidelines and their perception of home treatment with Miltefosine. Health facilities were assessed (structured spot inspection) for availability of diagnostic kits and drugs, records and equipment required to treat VL and side effects.

We compare means and proportions for various patient and provider characteristics for differences seen in 2009 and 2010 separately for each site. Due to small samples of VL patients and providers, we used Fischer's exact test to test for significance of differences not disaggregated by site.

## 3. Results

A total of 92 (India-63; Nepal-8; Bangladesh-21) new VL patients were identified through these active case detection strategies (camps, focal search around index case, etc.) in 2009 and 41 (India-8; Nepal-20; Bangladesh-13) in 2010. Thirteen newly diagnosed VL patients in 2009 from Bangladesh were thought to be cases of VL relapse and were referred to the district hospital for further treatment. We were not able to ascertain the treatment status of these patients as they were lost to follow-up. Patients were significantly younger (mean age 22 years, SD-16.47) in 2010 compared to 33 years (SD-15.9) in 2009 (*P* value = 0.009). The proportion of women was significantly higher (32% in 2010 compared to 8% in 2009; *P* value = 0.027).

### 3.1. VL Patient Experiences: Preintervention and Program Intervention Phase

Overall, the proportion of VL patients treated with Miltefosine as 1st line of treatment increased from 33% in 2009 to 59% in 2010 (Table 1) except in Nepal where it decreased from 38% to 20%. In contrast, use of SAG as primary treatment decreased from 53% in 2009 to 2% in 2010. Amphotericin B was the mainstay of treatment in Nepal (63% in 2009; 80% in 2010). One patient from India in 2009 received an incorrect dosage schedule of VL treatment. Furthermore, treatment was interrupted in 1 patient in India in 2009 and 1 each in India (due to side effects) and Nepal (due to drug shortage) in 2010. Overall, 3% and 10% patients experienced side effects in 2009 and 2010, respectively—the increase in minor side effects (diarrhea and vomiting) was seen to parallel increased use of Miltefosine. All patients reported that they received advice from their doctors regarding need to comply with treatment. In contrast, doctor's advice on possibility of drug side effects during treatment was received by only 8% of patients in 2009—this increased to 63% in 2010. A similar increase (61% in 2009 to 76% in 2010) was seen for receiving doctor's advice on need to comply with follow-up visits during treatment.

### 3.2. VL Treatment Practices and Infrastructure: Quality Aspects of Treatment Care

A total of 31 (India: 23; Nepal: 3; Bangladesh: 5) initial and 30 (India: 13; Nepal: 0; Bangladesh: 17) follow-up doctor consultations were observed in 2009 and 53 (India-23; Nepal-12; Bangladesh-18) initial and 20 (India-0; Nepal-0; Bangladesh-20) follow-up doctor consultations in 2010 (Table 2). Overall, doctors asked for family history of VL in about 68% of initial consultations in 2009 and 72% in 2010. Doctors in India were least likely (57% in 2009 and 48% in 2010) to ask for family history of VL in contrast to Bangladesh where all doctors asked for family history of VL. Only about 1 in 6 (17%) doctors in Nepal asked for history to rule out pregnancy before starting Miltefosine. In contrast, 71% (in 2009) and 100% (in 2010) of doctors in India ruled out pregnancy on history. Almost all doctors advised drug dosage based on the patient's weight and age. Measurement of patient's temperature and palpation for spleen size to reconfirm the initial diagnosis made by physicians at the case detection camps and for monitoring treatment recovery was done in 51% in 2009 and 63% and 73% in 2010, respectively. This was least likely in Bangladesh (23% in 2009 and 47% in 2010), while in India, this increased from 64% in 2009 to 100% in 2010. Doctors advised testing patient's blood for haemoglobin level in less than half of the consultations observed both in 2009 and 2010. Again, doctors in Bangladesh were least likely to advise testing during treatment (14% in 2009 increasing marginally to 32% in 2010). In Nepal, the proportion of doctors asking for family history of VL, advising drug dosage based on patient's weight, measurement of patient's temperature, and advice for testing blood during treatment was lower in 2010 compared to 2009. Overall, a substantial increase (47% in 2009 to 95% in 2010) was seen in doctors inquiring for side effects during follow-up visits. This increase was most marked in Bangladesh (6% in 2009, 95% in 2010).

Overall, patient satisfaction in all aspects of care increased significantly in 2010 compared to 2009 (Table 3) except for a marginal nonsignificant decrease of satisfaction in the amount of time provider spent with the patient. Patient satisfaction score was highest for general and technical aspects of care (4.11 in 2009; 4.43 in 2010), provider manners (3.19 in 2009; 4.25 in 2010) and level of provider communication (3.21 in 2009; 4.17 in 2010) but was lower for provider access (2.933 in 2009; 3.68 in 2010), and time spent by provider with the patient (3.70 in 2009; 3.56 in 2010). Patient satisfaction for general aspects of care, provider manners and communication, ease of provider access and time spent by provider, financial aspects of care was lower in Bangladesh in 2009 but became comparable with India and Nepal in 2010. 

**Figure 1:**
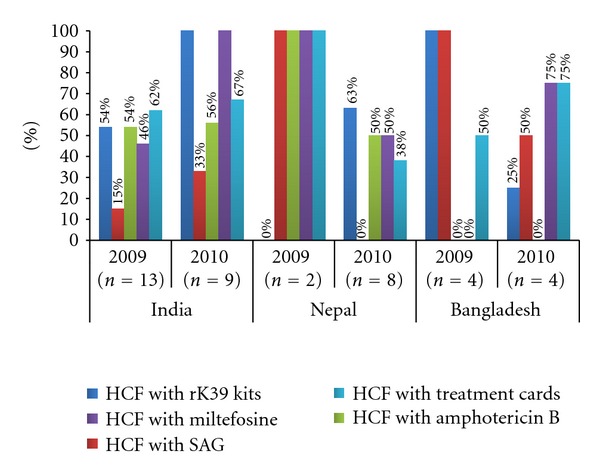
Health care facility (HCF) infrastructure to diagnose and treat VL.

Overall, health care facilities were better stocked with rK39 diagnostic kits in 2010 (9 of 9 and 5 of 8 health facilities inspected in India and Nepal, resp.) compared to 2009 (7 of 13 in India and 0 of 2 in Nepal) (Figure 1). In contrast, 4 of 4 health facilities had rK39 kits stocked in 2009 compared to only 1 of 4 in 2010 in Bangladesh. Similarly, Miltefosine stocks were available in 6 of 13 and 0 of 4 health facilities in 2009 compared to 9 of 9 and 3 of 4 health facilities in 2010 in India and Bangladesh, respectively. In contrast, 2 of 2 health facilities had Miltefosine stocks in 2009 compared to only 4 of 8 in 2010 in Nepal. None of the health facilities inspected in Bangladesh had stocks of Amphotericin B in both 2009 and 2010. Overall, there was no significant difference (*P* value = 0.349) in the proportion of health facilities keeping patient specific VL treatment cards in 2009 (63%) compared to 2010 (57%).

### 3.3. Provider Perspectives on VL Management and Adherence to Guidelines

A total of 30 (India: 19, Nepal: 7, and Bangladesh: 4) and 27 (India: 21, Nepal: 2, and Bangladesh: 4) physicians were interviewed in 2009 and 2010, respectively (Table 4). All physicians in Nepal and Bangladesh used rK39 test results as basis for starting VL treatment while in India 18 of 19 in 2009 and 13 of 21 physicians in 2010 used rK39 test to initiate VL treatment. Overall, Miltefosine was preferred for VL treatment by 73% of physicians in 2009 increasing to 93% in 2010. In India, 89% physicians in 2009 and 95% in 2010 reported Miltefosine use in their practice, while Amphotericin B use decreased from 58% in 2009 to 14% in 2010. Less than 50% physicians reported SAG use in both 2009 and 2010. In Nepal, physician use for Miltefosine and Amphotericin B was same (71% in 2009) (50% in 2010). In Bangladesh, physicians used antimonials (SAG) for VL treatment in 2009 and Miltefosine when it became available in 2010. Safety and availability of drug were the most important criteria for physician's choice of drug for VL treatment in all sites. In Bangladesh, affordability and effectiveness of the drug were equally important criteria for physician's drug choice. In India, 63% physicians in 2009 and 57% in 2010 reported they advised blood tests before or during treatment. In Bangladesh, this proportion fell from 100% in 2009 to 50% in 2010. The most common reasons for not advising blood tests were either because the physician felt there was no need or there was no laboratory technician available to do the blood tests. In Nepal, all physicians reported advising a blood test during VL treatment. Overall, physician awareness of side effects of Miltefosine was lower in Bangladesh. Physician awareness of diarrhea or vomiting was high in India (100%) and Nepal (71% in 2009 and 100% in 2010). Physician awareness of other side effects of Miltefosine like jaundice (0%) and teratogenicity (40%) was lower in 2009 and increased marginally in 2010. A higher proportion of physicians recommended home-based treatment with Miltefosine in 2010 (93%) compared to 2009 (60%) except in Nepal where 1 of 2 (50%) physicians recommended home treatment in 2010 compared to 5 of 7 (71%) in 2009. Almost all physicians in India, Nepal, and Bangladesh in 2010 felt that Miltefosine treatment under direct observation at home would be effective.

Despite Miltefosine being available free of cost to the patient through the program, 12% of the health workers interviewed in Bangladesh perceived the cost of VL treatment to be affordable, while 18% perceived it to be too high. A majority of health staff felt that lack of patient awareness and lack of access to facility (100% in India; 82% in Bangladesh) were major challenges to early detection and diagnosis of VL. Drug shortage and lack of patient compliance were perceived by health workers to be major barriers to VL treatment in India. About half of all health workers in India and Bangladesh reported cost as a barrier to VL treatment. In India, 64% of health workers reported occurrence of drug-related side effects as a problem for VL treatment compared to 18% of health workers in Bangladesh.

## 4. Discussion

In the preintervention phase (2009), VL patients were diagnosed using rK39 tests conducted by the researchers. These newly diagnosed VL cases were then referred to health centers and their further treatment was ensured by the researchers. In contrast, in 2010, the researchers' role was restricted to training of trainers in the health system in home-based VL management with Miltefosine applying national treatment guidelines. Diagnosis and home-based treatment of VL cases under direct observation of a health functionary or volunteer in 2010 was fully the responsibility of the health system (program intervention phase). The researchers assessed the availability of diagnostics and drugs in peripheral health facilities and evaluated VL case management as implemented by the health system.

The use of antimonials showed a dramatic decline in 2010 as preference shifted towards Miltefosine as the primary line of VL treatment—in Bangladesh, this shift was consequent to introduction of Miltefosine in the country while in India it was more due to its availability in the public sector at no cost to the patient. In Nepal, at the time of the study Amphotericin B continued to be used as the drug of choice for VL treatment in 2010 as one of the district hospital had to dispense a large stock before its expiry. Also, Amphotericin B was preferred by physicians at one of the zonal hospitals.

The shift towards Miltefosine was paralleled by an increase in the known side effects of Miltefosine (diarrhea and vomiting) but a decrease in side effects from antimonials (not documented in this study). Almost all patients received the correct dosage schedule in both study phases indicating correct prescription practices and a high level of knowledge about drug dosage and schedules amongst physicians. Treatment was interrupted in less than 5% of patients in either phase (in one patient due to drug shortage). Miltefosine dosage schedule is based on the patient's age and body weight. Its use is contraindicated in infants, pregnant, or breast-feeding women or women of reproductive age group unless counselled to prevent pregnancy with use of oral contraceptives [12]. A pregnancy test is also generally recommended to rule out pregnancy prior to Miltefosine use. Medical supervision is advised twice a week and monitoring for hepatic and renal function is recommended wherever feasible according to national guidelines. Counselling for need to comply with full treatment seemed to be adequate in both study phases. Counselling for need to follow up weekly during treatment was lower in 2009 but increased in the postintervention phase especially in India. Counselling of patients on potential side effects was still inadequate and needs to be strengthened. Only about 1 in 6 physicians in Nepal ruled out pregnancy on history in women of reproductive age. Pregnancy test or contraceptive counselling was not done for any woman of reproductive age prior to Miltefosine use. The quality of care given by providers was inadequate. Fever was measured in less than half of initial or follow-up visits in Nepal and Bangladesh. Spleen size was assessed by palpation in less than half of initial or follow-up visits in Bangladesh. Blood tests (biochemical monitoring before and during treatment) were advised in less than half of all initial or follow-up visits in Nepal and Bangladesh. Monitoring of side effects during follow-up visits was almost universal in the postintervention phase. Though there was a significant improvement in patient satisfaction levels in the postintervention phase, patients were less satisfied with ease of access to provider and the time spent by the provider with them. Generally patients in India were more satisfied with how providers communicated with them as well as their manners and attitudes in the postintervention phase compared to patients in Nepal and Bangladesh. However, these differences in satisfaction levels could be cultural based on patient's experiences and expectations of health services. Overall, health facilities were better stocked with rK39 diagnostic kits and VL drugs in 2010 except in Bangladesh where 3 of the 4 health facilities inspected did not have rK39 diagnostic kits. Drug supply was available and adequate in health facilities in India. Amphotericin B as 2nd line of treatment was not available in more than half of all health facilities in India and Nepal and not at all available in Bangladesh. Drug supply needs to be strengthened especially in Nepal and Bangladesh. The maintenance of patient specific treatment cards needs to be strengthened at all health facilities in each country.

In summary, physician counselling practices requires to be strengthened. Despite availability, about one-third of physicians (especially in the private sector) in India still rely on tests other than rK39 for VL diagnosis. Physician choice is driven by the availability of the drug and its perceived safety and less so by affordability or effectiveness of the drug. Ensuring an adequate drug supply will greatly influence physician prescription practice for VL. Physician awareness of the common side effects of Miltefosine like diarrhea and vomiting is high. However, awareness about important but less common side effects of Miltefosine like liver and renal toxicity is low especially in Bangladesh and needs to be improved. Testing blood for haemoglobin, liver, and renal function before and during VL treatment is still not routine practice with physicians in India and Bangladesh. Improving physician awareness of potential side effects of Miltefosine and ensuring availability of laboratory services will strengthen physician practice of routine bio-chemical monitoring before and during VL treatment. National guidelines stipulate that Miltefosine can be administered at home under direct supervision of a health volunteer/health functionary. Though almost all physicians in India, Nepal, and Bangladesh feel that home-based treatment of VL with Miltefosine is effective, only about half the physicians in Nepal would recommend it.

## 5. Conclusion

Though limited by small samples of patients interviewed and doctor-patient consultations observed and health facilities inspected, our study provides evidence on constraints and prospects of VL management in VL endemic districts of India, Nepal, and Bangladesh when implemented by national VL elimination programs. This underlines the need for continued operational research to direct the Kala-azar elimination programme.

## Figures and Tables

**Table 1:** VL case management: patient experiences.

	India		Nepal		Bangladesh		Overall	
	2009	2010	2009	2010	2009	2010	2009	2010
No. of new VL cases detected	63	8	8	20	21^2^	13	92	41
No. of VL cases started with (%)								
(i) SAG	34/63 (54%)	1/8 (13%)	0/8 (0%)	0/20 (0%)	8/8 (100%)	0/13 (0%)	42/79 (53%)	1/41 (2%)
(ii) Amphotericin B	6/63 (10%)	0/8 (0%)	5/8 (63%)	16/20 (80%)	0/8 (0%)	0/13 (0%)	11/79 (14%)	16/41 (39%)
(iii) Miltefosine	23/63 (37%)	7/8 (88%)	3/8 (38%)	4/20 (20%)	0/8 (0%)	13/13 (100%)	26/79 (33%)	24/41 (59%)
No. of VL cases given correct dosage schedule (%)	62/63 (98%)	8/8 (100%)	3/3 (100%)	20/20 (100%)	8/8 (100%)	13/13 (100%)	73/74 (99%)	41/41 (100%)
No. of VL cases with treatment interrupted (%)	1/63 (2%)	1/8 (13%) (side effect)	0/3 (0%)	1/20 (5%) (no drug)	0/8 (0%)	0/13 (0%)	1/74 (1%)	2/41 (5%)
No. VL cases experiencing side effects (%)	2/63 (3%)	1/8 (13%)	0/3 (0%)	1/20 (5%)	0/8 (0%)	2/13 (15%)	2/74 (3%)	4/41 (10%)
No. VL cases received doctor's advice on (%)								
(i) Need to comply with treatment (%)	63/63 (100%)	8/8 (100%)	3/3 (100%)	20/20 (100%)	—^3^	13/13 (100%)	66/66 (100%)	41/41 (100%)
(ii) Side effects (%)	2/63 (3%)	8/8 (100%)	3/3 (100%)	6/20 (30%)	—^2^	12/13 (92%)	5/66 (8%)	26/41 (63%)
(iii) Compliance with follow up (%)	37/63 (59%)	8/8 (100%)	3/3 (100%)	11/20 (55%)	—^2^	12/13 (92%)	40/66 (61%)	31/41 (76%)
Did you comply with FU advise	37/63 (59%)	8/8 (100%)	3/3 (100%)	7/20 (35%)	—^2^	12/13 (92%)	40/66 (61%)	27/41 (66%)
Volunteer support for home treatment with Miltefosine (%)	15/23 (65%)	4/7 (57%)	3/3 (100%)	3/4 (75%)	NA^4^	4/13 (31%)	18/26 (69%)	11/24 (46%)

^2^13 patients were diagnosed as relapse of VL and referred to district hospital. Treatment status could not be ascertained subsequently.

^3^Information on doctor advice to patients is not captured in Bangladesh in the interview for patients who received other than Miltefosine treatment.

^4^Not applicable as no patient received Miltefosine.

**Table 2:** VL management practices: observation of doctor patient consultations.

	India		Nepal		Bangladesh		Overall	
	2009	2010	2009	2010	2009	2010	2009	2010
No. of doctor-patient consultations observed: 1st visit	(*n* = 23)	(*n* = 23)	(*n* = 3)	(*n* = 12)	(*n* = 5)	(*n* = 18)	(*n* = 31)	(*n* = 53)
Follow-up visit	(*n* = 13)	(*n* = 0)	(*n* = 0)	(*n* = 0)	(*n* = 17)	(*n* = 20)	(*n* = 30)	(*n* = 20)

(i) Doctor asked patient for family h/o VL at 1st visit	13/23 (57%)	11/23 (48%)	3/3 (100%)	9/12 (75%)	5/5 (100%)	18/18 (100%)	21/31 (68%)	38/53 (72%)
(ii) Doctor asked about pregnancy at 1st visit of women of reproductive age	5/7 (71%)	6/6 (100%)	—	1/6 (17%)	1/1 (100%)	—	6/8 (75%)	7/12 (58%)
(iii) Doctor advised drug dose based on patient's weight at 1st visit	22/23 (96%)	23/23 (100%)	3/3 (100%)	10/12 (83%)	5/5 (100%)	18/18 (100%)	30/31 (97%)	51/53 (96%)
(iv) Temperature of patient was measured	23/36 (64%)	23/23 (100%)	3/3 (100%)	5/12 (42%)	5/22 (23%)	18/38 (47%)	31/61 (51%)	46/73 (63%)
(v) Doctor examined patient for spleen enlargement	23/36 (64%)	23/23 (100%)	3/3 (100%)	12/12 (100%)	5/22 (23%)	18/38 (47%)	31/61 (51%)	53/73 (73%)
(vi) Doctor advised blood tests for patient	21/36 (58%)	20/23 (87%)	3/3 (100%)	5/12 (42%)	3/22 (14%)	12/38 (32%)	27/61 (44%)	37/73 (51%)
(vii) On FU visit, doctor asked patient for side effects	13/13 (100%)	—	—	—	1/17 (6%)	19/20 (95%)	14/30 (47%)	19/20 (95%)

**Table 3:** Patient satisfaction with medical care (significant differences in patient satisfaction scores between 2009 and 2010 in boldface).

	India		Nepal		Bangladesh		Overall	
	2009	2010	2009	2010	2009	2010	2009	2010
	(*n* = 60)	(*n* = 8)	(*n* = 3)	(*n* = 22^5^)	(*n* = 8)	(*n* = 13)	(*n* = 71)	(*n* = 43)
Mean patient satisfaction score (SD)								
(i) General aspects of care	4.20 (0.944)	4.56 (0.903)	4.50 (0.000)	4.38 (0.858)	**3.31 ** (0.530)	**4.42 ** (0.534)	4.11 (0.931)	4.43 (0.768)
(ii) Technical aspects of care	**3.62 ** (0.557)	**4.09 ** (0.421)	**3.41 ** (0.144)	**4.37 ** (0.538)	**3.71 ** (0.507)	**4.23 ** (0.494)	**3.62 ** (0.539)	**4.27 ** (0.506)
(iii) Provider manner	**3.32 ** (0.573)	**4.62 ** (0.517)	**3.33 ** (0.577)	**4.15 ** (0.543)	**2.12 ** (0.991)	**4.19 ** (0.830)	**3.19 ** (0.728)	**4.25 ** (0.648)
(iv) Level of provider communication	3.20 (0.576)	3.87 (1.093)	4.33 (0.288)	4.25 (0.550)	**2.87 ** (0.640)	**4.23 ** (0.632)	**3.21 ** (0.624)	**4.17 ** (0.697)
(v) Financial aspects of care	**2.61 ** (0.820)	**3.31 ** (0.593)	3.83 (0.288)	4.02 (0.892)	**2.31 ** (1.412)	**3.57 ** (0.812)	**2.63 ** (0.917)	**3.75 ** (0.854)
(vi) Time spent by provider	3.96 (1.149)	3.93 (0.821)	**2.16 ** (0.288)	**3.36 ** (0.639)	**2.31 ** (0.593)	**3.69 ** (0.878)	3.70 (1.238)	3.56 (0.768)
(vii) Ease of provider access	**3.05** (0.590)	**3.59 ** (0.376)	3.7 (0.000)	3.88 (0.413)	**1.68 ** (0.513)	**3.38 ** (0.976)	**2.93 ** (0.734)	**3.68 ** (0.657)

^5^Includes 2 nonstudy VL patients.

**Table 4:** Physician perspectives of VL management.

	India		Nepal		Bangladesh		Overall	
	2009	2010	2009	2010	2009	2010	2009	2010
No. of physicians interviewed	(*n* = 19)	(*n* = 21)	(*n* = 7)	(*n* = 2)	(*n* = 4)	(*n* = 4)	(*n* = 30)	(*n* = 27)
Rk39 test as decision criteria to treat VL	18/19 (95%)	13/21 (62%)	7/7 (100%)	2/2 (100%)	4/4 (100%)	4/4 (100%)	29/30 (97%)	19/27 (70%)
Physician's use of VL drug								
(i) SAG	8/19 (42%)	10/21 (48%)	0/7 (0%)	0/2 (0%)	4/4 (100%)	4/4 (100%)	12/30 (40%)	14/27 (52%)
(ii) Amphotericin B	11/19 (58%)	3/21 (14%)	5/7 (71%)	1/2 (50%)	0/4 (0%)	0/4 (0%)	16/30 (53%)	4/27 (15%)
(iii) Miltefosine	17/19 (89%)	20/21 (95%)	5/7 (71%)	1/2 (50%)	0/4 (0%)	4/4 (100%)	22/30 (73%)	25/27 (93%)
Physician criteria for VL drug selection								
(i) Available	14/19 (74%)	19/21 (90%)	4/7 (57%)	2/2 (100%)	4/4 (100%)	3/4 (75%)	22/30 (73%)	24/27 (89%)
(ii) Affordable	8/19 (42%)	11/21 (52%)	1/7 (14%)	0/2 (0%)	4/4 (100%)	3/4 (75%)	13/30 (43%)	14/27 (52%)
(iii) Safe	18/19 (95%)	21/21 (100%)	4/7 (57%)	1/2 (50%)	4/4 (100%)	3/4 (75%)	26/30 (87%)	25/27 (93%)
(iv) Effective	12/19 (63%)	12/21 (57%)	4/7 (57%)	1/2 (50%)	4/4 (100%)	3/4 (75%)	20/30 (67%)	16/27 (59%)
No. physician advises blood tests (%)?	12/19 (63%)	12/21 (57%)	7/7 (100%)	2/2 (100%)	4/4 (100%)	2/4 (50%)	23/30 (77%)	16/27 (59%)
Reason for not advising blood tests?								
No need	4/7 (57%)	—	—	—	—	1/2 (50%)	4/7 (57%)	1/11 (9%)
No lab technician	—	9/9 (100%)	—	—	—	1/2 (50%)	—	10/11 (91%)
No. physician aware of Miltefosine side effects (%)								
(i) Diarrhea	19/19 (100%)	21/21 (100%)	5/7 (71%)	2/2 (100%)	0/4 (0%)	0/4 (0%)	24/30 (80%)	23/27 (85%)
(ii) Vomiting	19/19 (100%)	21/21 (100%)	5/7 (71%)	2/2 (100%)	0/4 (0%)	3/4 (75%)	24/30 (80%)	26/27 (96%)
(iii) Jaundice	0/19 (0%)	12/21 (57%)	0/7 (0%)	1/2 (50%)	0/4 (0%)	1/4 (25%)	0/30 (0%)	14/27 (52%)
(iv) Abdominal pain	5/19 (26%)	9/21 (43%)	1/7 (14%)	2/2 (100%)	0/4 (0%)	3/4 (75%)	6/30 (20%)	14/27 (52%)
(v) Renal toxicity	14/19 (74%)	4/21 (19%)	2/7 (29%)	1/2 (50%)	0/4 (0%)	0/4 (0%)	16/30 (53%)	5/27 (19%)
(vi) Teratogenicity	11/19 (58%)	11/21 (52%)	1/7 (14%)	1/2 (50%)	0/4 (0%)	0/4 (0%)	12/30 (40%)	12/27 (44%)
No. physician recommend home based Miltefosine treatment (%)	10/19 (53%)	20/21 (95%)	5/7 (71%)	1/2 (50%)	3/4 (75%)	4/4 (100%)	18/30 (60%)	25/27 (93%)
No. physician feel home-based Miltefosine treatment is effective (%)?	15/19 (79%)	20/21 (95%)	6/7 (86%)	2/2 (100%)	1/4 (25%)	4/4 (100%)	22/30 (73%)	26/27 (96%)
